# Circular RNA circ_0006168 enhances Taxol resistance in esophageal squamous cell carcinoma by regulating miR-194-5p/JMJD1C axis

**DOI:** 10.1186/s12935-021-01984-y

**Published:** 2021-05-22

**Authors:** Fanyong Qu, Lina Wang, Caiyan Wang, Lingxia Yu, Kaikai Zhao, Hao Zhong

**Affiliations:** 1 Department of Radiation Oncology, Yantai Affiliated Hospital of Binzhou Medical University, No. 717, Jinbu Street, Mu ping District, Yantai, Shandong 264100 China; 2 Department of Oncology, Yantai Affiliated Hospital of Binzhou Medical University, Yantai, Shandong 264100 China; 3 Department of Gastroenterology, Yantai Affiliated Hospital of Binzhou Medical University, Yantai, Shandong 264100 China

**Keywords:** Esophageal squamous cell carcinoma, Taxol resistance, circ_0006168, miR-194-5p, JMJD1C

## Abstract

**Background:**

Chemoresistance is one of the major obstacles for cancer therapy in the clinic. Circular RNAs (circRNAs) are involved in the pathogenesis of esophageal squamous cell carcinoma (ESCC) and chemoresistance. This study aimed to explore the role and molecular mechanism of circ_0006168 in Taxol resistance of ESCC.

**Methods:**

The expression levels of circ_0006168, microRNA-194-5p (miR-194-5p) and jumonji domain containing 1C (JMJD1C) were measured by quantitative real-time polymerase chain reaction (qRT-PCR) or western blot. The half-inhibition concentration (IC_50_) value of Taxol was evaluated by Cell Counting Kit-8 (CCK-8) assay. Cell proliferation was evaluated by CCK-8 and colony formation assays. Cell migration and invasion were detected by transwell assay. Cell apoptosis was determined by flow cytometry. The interaction between miR-194-5p and circ_0006168 or JMJD1C was predicted by bioinformatics analysis (Circinteractome and TargetScan) and verified by dual-luciferase reporter and RNA Immunoprecipitation (RIP) and RNA pull-down assays. The mice xenograft model was established to investigate the roles of circ_0006168 in vivo.

**Results:**

Circ_0006168 and JMJD1C were upregulated and miR-194-5p was downregulated in ESCC tissues, ESCC cells, and Taxol-resistant cells. Functionally, knockdown of circ_0006168 or JMJD1C increased Taxol sensitivity of ESCC in vitro via inhibiting cell proliferation, migration and invasion, and promoting apoptosis. Moreover, circ_0006168 could directly bind to miR-194-5p and JMJD1C was verified as a direct target of miR-194-5p. Mechanically, circ_0006168 was a sponge of miR-194-5p to regulate JMJD1C expression in ESCC cells. Furthermore, JMJD1C overexpression reversed the promotive effect of circ_0006168 knockdown on Taxol sensitivity. Besides, circ_0006168 silence suppressed tumor growth in vivo.

**Conclusion:**

Circ_0006168 facilitated Taxol resistance in ESCC by regulating miR-194-5p/JMJD1C axis, providing a promising therapeutic target for ESCC chemotherapy.

**Supplementary Information:**

The online version contains supplementary material available at 10.1186/s12935-021-01984-y.

## Introduction

Esophageal cancer (EC) is a common malignant tumor worldwide, ranking the 7th in incidence (approximately 572,000 new cases) and 6th in mortality (approximately 509,000 deaths) [1, 2]. Esophageal squamous cell carcinoma (ESCC) is the most common histological type in EC and nearly half of these ESCC cases have occurred in China [3]. Although surgery, radiation therapy, chemotherapy and molecular targeted therapy have all made significant contributions to treatment, the 5 year survival rate for ESCC patients is still poor because patients are frequently diagnosed at advanced stages [4, 5]. Besides, chemotherapy resistance (including Taxol resistance) is also an essential factor leading to poor prognosis in ESCC patients [6, 7]. Therefore, it is essential to explore the mechanism of chemotherapy resistance or sensitivity for improving the prognosis of ESCC.

Circular RNA (circRNA) are a novel type of noncoding RNAs (ncRNAs) with covalently closed-loop structures lacking 5’-cap and 3’-end poly A tail [8, 9]. Due to the special circular structures, circRNAs are not influenced by RNA excision enzymes and more stable than linear RNAs [10]. In recent years, growing evidence has indicated that circRNAs can function as pivotal mediators in modulating the progression of diverse cancers, including ESCC [11]. For instance, circRNA circ-TTC17 acted as a tumor suppressor through repressing ESCC cell growth and migration [12]. Moreover, circRNA hsa_circ_0000337 served as a tumor promoter in ESCC via promoting cell proliferation, migration and invasion [13]. In addition, circRNAs have been reported to play critical roles in Taxol resistance [14, 15]. Hsa_circ_0006168 (circ_0006168) is derived from CCR4-NOT transcription complex subunit 6 like (CNOT6L) gene and located at chr4:78,694,234–78,697,546, and it has been reported to act as an oncogene in ESCC [16]. Moreover, hsa_circ_0030998 suppressed lung cancer tumorigenesis and Taxol resistance by sponging miR‐558 [17]. In addition, hsa_circ_0002483 inhibited the progression and enhanced the Taxol sensitivity of non-small cell lung cancer via regulating miR-182-5p [18]. Nevertheless, the exact roles and regulatory mechanism of circ_0006168 in Taxol resistance of ESCC remain largely unknown.

CircRNAs are identified as competing endogenous RNAs (ceRNAs) or microRNA (miRNA) sponges/decoys in many cancers [19]. MiR-194-5p, an endogenous miRNA with low expression in many cancers, was shown to suppress ESCC cell progression [20, 21]. Moreover, miR-194-5p downregulation induced Taxol resistance in ovarian cancer cells via affecting MDM2 expression [22]. Jumonji domain containing 1C (JMJD1C) has been identified as a DNA-damage response (DDR) component to regulate the cellular behaviors of cancer cells [23]. A previous research has demonstrated that JMJD1C can promote EC cell proliferation [24]. Base on the analysis of bioinformatics software (Circinteractome and TargetScan), we found that circ_0006168 and JMJD1C had complementary binding sequence for miR-194-5p, which stimulated us to assume the ceRNA regulatory network of circ_0006168/ miR-194-5p/JMJD1C in Taxol resistance of ESCC.

In this work, the abundance of circ_0006168, miR-194-5p and JMJD1C in ESCC tissues, ESCC cells, and Taxol-resistant cells was determined. Then we investigated the biological roles of circ_0006168 in Taxol-resistant cell growth, migration, invasion, and apoptosis. Additionally, the function of circ_0006168 in vivo was investigated via xenograft tumor model. The purpose of this research was to offer a promising therapy strategy for ESCC with Taxol resistance.

## Materials and methods

### Specimen collection

In this study, ESCC tissues (n = 40) and adjacent normal tissues (n = 40) were acquired from patients who had undergone surgery at Yantai Affiliated Hospital of Binzhou Medical University. None of the patients with ESCC received systemic or local treatment before the operation. These tissues were fast-frozen in liquid nitrogen and preserved at -80℃ until required. Informed consents have been obtained from all patients. And the protocols have been approved by the Research Ethics Committee of Yantai Affiliated Hospital of Binzhou Medical University.

### Cell culture and transfection

Human oesophageal epithelial cells (HET-1A) and ESCC cells (Eca109) were purchased from Fenghuishengwu (Wuhan, China), and ESCC cells (KYSE450, KYSE150 and KYSE30) were bought from COBIOER (Nanjing, China). The cell culture medium was the RPMI-1640 medium (Invitrogen, Carlsbad, CA, USA) supplemented with 10% fetal bovine serum (FBS; Invitrogen). All cells were maintained in a 5% CO_2_ incubator at 37℃. Eca109 and KYSE150 cells were exposed to increasing doses of Taxol (Solarbio, Beijing, China) to establish Taxol-resistant ESCC cell lines (Eca109/Taxol and KYSE150/Taxol). Then, Taxol-resistant cells were treated with Taxol (10 nM) to maintain a resistant phenotype.

The small interfering RNA (siRNA) targeting circ_0006168 or JMJD1C (si-circ_0006168 or si-JMJD1C) and matched control (si-control), pcDNA-JMJD1C overexpression plasmid (pcDNA-JMJD1C) and matched control (pcDNA), miR-194-5p mimic (miR-194-5p) and matched control (miR-NC), miR-194-5p inhibitor (inhibitor-miR-194-5p) and matched control (inhibitor-NC) were obtained from Genecreat (Wuhan, China). Lentivirus-mediated short hairpin RNA (shRNA) targeting circ_0006168 (sh-circ_0006168) and corresponding control (sh-control) were provided by Genechem (Shanghai, China). Transient transfection was carried out in our research by using Lipofectamine 3000 Reagent (Invitrogen).

### RNA isolation and quantitative real-time polymerase chain reaction (qRT-PCR)

Total RNA was extracted using TRIzol reagent (Invitrogen). Subsequently, complementary DNA (cDNA) was reverse-transcribed using miRNA cDNA synthesis kit (Ribobio, Guangzhou, China) for miRNA and PrimeScriptTM RT reagent Kit (TaKaRa, Dalian, China) for mRNA and circRNA. Next, SYBR qPCR Master Mix (Vazyme, Nanjing, China) was adopted for qRT-PCR analysis on CFX96 Touch Real-Time PCR Detection System (Bio-Rad, Hercules, CA, USA). The primers and their sequences were listed as follows: circ_0006168 (sense, 5’-ACCAGCAGAACTAGGAAACA-3’; anti-sense, 5’-TGGCATCCCTATTAGTCTTTC-3’); CNOT6L (sense, 5’-GGTAGAGTGCGGAGCCTAAG-3’; anti-sense, 5’-CAGGTGGAATGCGACTAAGGT-3’); miR-194-5p (sense, 5’- GGGTGTAACAGCAACTCCA -3’; anti-sense, 5’-CAGTGCGTGTCGTGGAGT-3’); JMJD1C (sense, 5’-TCCTGTCAGACCTTCCAGTGCA-3’; anti-sense, 5’-GTGGATGCAACAGACCGTAATGG-3’); glyceraldehyde-3-phosphate dehydrogenase (GAPDH) (sense, 5’-GGGAAGCTCACTGGCATGGCCTTCC-3’; anti-sense, 5’-CATGTGGGCCATGAGGTCCACCAC-3’); U6 (sense, 5’- CTCGCTTCGGCAGCACATATACT-3’; anti-sense, 5’-ACGCTTCACGAATTTGCGTGTC-3’). Gene expression was detected according to the 2-^ΔΔCt^ method. GAPDH (for circ_0006168, CNOT6L and JMJD1C) and U6 (for miR-194-5p) were acted as the internal references.

### RNase R treatment

To determine the circular characteristic of circ_0006168, total RNA (2 µg) was incubated with or without 6 units of RNase R (Geneseed Biotech, Guangzhou, China) for 0.5 h at 37℃. Subsequently, the expression of circ_0006168 and JMJD1C mRNA was tested via qRT-PCR analysis.

### Cell counting Kit-8 (CCK-8) assay

CCK-8 purchased from Boster (Wuhan, China) was used for analyzing cell viability and half-inhibition concentration (IC_50_) value of Taxol. In brief, 100 uL cell suspension (2 × 10^3^ cells) was added into a 96-well plate. CCK-8 reagent (10 µL) was added to per well after respective treatment. After incubation for 2–3 h, a microplate reader (Bio-Rad) was employed to examine the absorbance of per well at 450 nm. IC_50_ value of Taxol was calculated using Graphpad Prism (GraphPad Software, San Diego, CA, USA).

### Colony formation assay

Briefly, transfected cells (Eca109/Taxol and KYSE150/Taxol) were inoculated in a six-well plate and the medium was updated every 3 days. After incubation for 2 weeks, the cells were fixed using the paraformaldehyde (4%, Beyotime, Jiangsu, China), followed by staining with crystal violet (0.1%, Sinopharm Chemical Reagent, Beijing, China). Next, microscope (Leica, Wetzlar, Germany) was used to count the number of colonies (> 50 cells per colony).

### Transwell assay

Transwell assay was used for measuring migration and invasion of Eca109/Taxol and KYSE150/Taxol cells. For migration, transfected cells (Eca109/Taxol and KYSE150/Taxol) suspended in serum-free RPMI-1640 (0.2 mL) were seeded into the top chamber of the 24-well transwell plates (Costar, Corning, NY, USA). The bottom chamber was filled with medium containing 10% FBS (0.6 mL). After 24 h transfection, the migrated cells fixed using the paraformaldehyde (4%, Beyotime) and stained by crystal violet (0.1%, Sinopharm Chemical Reagent). The images were captured under a microscope (Leica) at × 100 magnification. Similar to the migration assay, excluded cells were seeded into a Matrigel-coated (BD Biosciences, Franklin, NJ, USA) top chamber for the invasion assay.

### Cell apoptosis assay

Annexin V-fluorescein isothiocyanate (FITC)/propidium iodide (PI) apoptosis detection kit (Keygen, Nanjing, China) was applied for detecting cell apoptosis. In short, Eca109/Taxol and KYSE150/Taxo cells were collected following transfection for 48 h, and then dyed with Annexin V-FITC and PI. After incubation for 20 min in the darkness, the percentage of apoptotic cells was examined via a flow cytometer (BD Biosciences).

### Dual-luciferase reporter assay

Potential binding sites of miR-194-5p and circ_0006168 or JMJD1C were provided by Circinteractome (https://circinteractome.nia.nih.gov/) and TargetScan (http://www.targetscan.org/). The fragments of circ_0006168 and JMJD1C 3’UTR that contained miR-194-5p binding sites were synthesized and inserted into pmirGlO luciferase reporter vector (Promega, Madison, WI, USA), namely wild-type reporter vectors (circ_0006168-wt and JMJD1C-wt). Meanwhile, their mutated-type reporter vectors (circ_0006168-mut and JMJD1C-mut) without binding sites were constructed in the same way. Each of the above-mentioned vectors was respectively transfected into Eca109/Taxol and KYSE150/Taxol cells along with miR-194-5p or miR-NC for 48 h. The luciferase activity was estimated through a Dual-luciferase Reporter Assay System (Promega).

### RNA immunoprecipitation (RIP) assay

RIP assay was carried out using an EZ-Magna RIP kit (Millipore, Billerica, MA, USA). Briefly, RIP lysis buffer was employed to lyse Eca109/Taxol and KYSE150/Taxol cells, and RIP buffer that contained magnetic beads conjugated with human antibody against anti-Ago2 or anti-IgG was utilized to incubate the lysate products. Next, the beads were digested with protease K, and circ_0006168, miR-194-5p and JMJD1C levels were detected using qRT-PCR analysis.

### RNA pull-down assay

Input negative control (NC-input), biotinylated negative control (bio-NC), biotinylated miR-194-5p (bio-miR-194-5p), and miR-194-5p-input were acquired from Santa Cruz Biotechnology (Dallas, Texas, USA) and transfected into Eca109/Taxol and KYSE150/Taxol cells. Dynabeads M-280 Streptavidin (Invitrogen) was used to incubate cell lysates. Lastly, circ_0006168 and JMJD1C levels were measured by qRT-PCR.

### Western blot assay

Total protein was isolated using RIPA lysis buffer (Solarbio) and then quantified using a BCA protein assay kit (Solarbio). Total protein (50 μg) was separated using sodium dodecyl sulfate–polyacrylamide gel electrophoresis (SDS-PAGE; Beyotime). After that, the separated proteins were transferred onto nitrocellulose membranes (Invitrogen). These membranes were incubated for 12 h at 4℃ using primary antibody against JMJD1C (ab130922, 1:2000, Abcam, Cambridge, UK) or GAPDH (ab37168, 1:1000, Abcam) after blocking with 5% non-fat milk. Afterwards, these membranes were continuously incubated by secondary antibody (ab205718, 1:4000, Abcam). At last, the enhanced chemiluminescence detection reagent (Applygen, Beijing, China) was utilized to visualize the protein bands.

### Tumor formation assay in vivo

BALB/c nude mice (n = 20, male, 5–6 weeks old, weighing 18–25 g) were provided by Vital River Laboratory Animal Technology Co., Ltd (Beijing, China). Eca109/Taxol and KYSE150/Taxol cells stably transfected with downregulating lentivirus (sh-circ_0006168) or control lentivirus (sh-NC) were subcutaneously injected into BALB/c nude mice (n = 5/group). The tumor volume was tested with a caliper every week and calculated as follows: 0.5 × length × width^2^. All mice would be sacrificed after injection for 5 weeks, and the tumor tissues were excised, weighed and harvested for detecting the abundance of circ_0006168, miR-194-5p and JMJD1C. This in vivo experiments were permitted by the Animal Care and Use Committee of Yantai Affiliated Hospital of Binzhou Medical University.

### Statistical analysis

Experimental data were presented by mean ± standard deviation (SD). Every test was executed at least three times. GraphPad Prism 7.0 (GraphPad Software Inc, La Jolla, CA, USA) was used to display the analysis of results. Student’s *t*-test and one-way analysis of variance (ANOVA) were performed to analyze significant differences between the groups. Statistical significance was considered when *P* < 0.05. *P < 0.05, **P < 0.001, ***P < 0.0001.

## Results

### Circ_0006168 was upregulated in ESCC tissues and cells, and Taxol-resistant cells

**Fig. 1:**
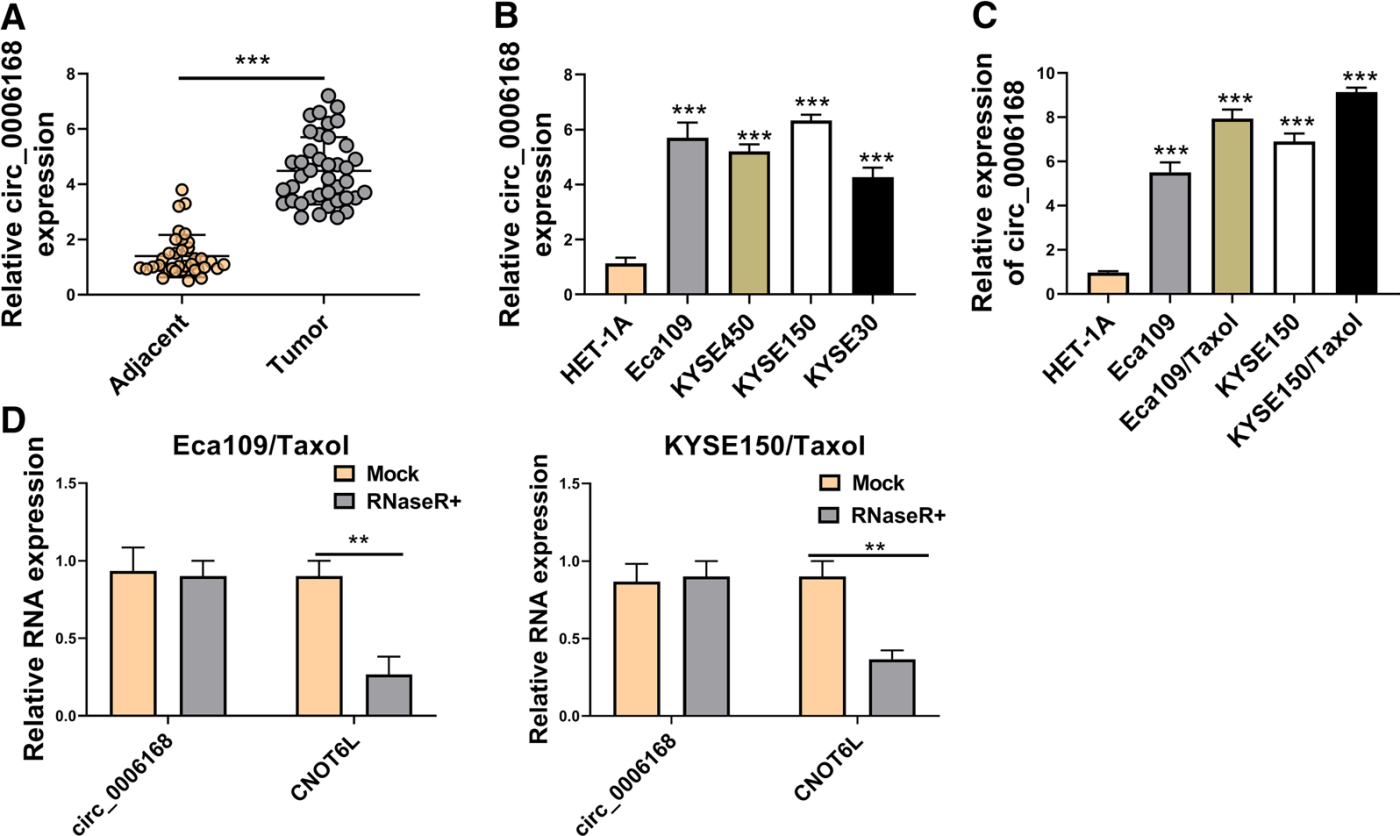
Circ_0006168 expression was increased in ESCC tissues, ESCC cells, and Taxol-resistant cells. **A** The expression level of circ_0006168 in ESCC tissues (n = 40) and adjacent normal tissues (n = 40) was detected by qRT-PCR. **B** The abundance of circ_0006168 in ESCC cells (Eca109, KYSE450, KYSE150, and KYSE30) and HET-1A cells was analyzed by qRT-PCR. **C** Circ_0006168 level was examined by qRT-PCR in HET-1A, Eca109, KYSE150 cells, Eca109/Taxol, and KYSE150/Taxol cells. **D** The expression levels of circ_0006168 and linear CNOT6L were determined after treatment of RNase R by qRT-PCR. **P < 0.001, ***P < 0.0001

To explore the potential roles of circ_0006168 in ESCC, the expression of circ_0006168 was detected by qRT-PCR in ESCC tissues and cells. Our study found that circ_0006168 expression in ESCC tissues was obviously higher than that in adjacent normal tissues (Fig. 1A). Likewise, compared with HET-1A cells, the expression of circ_0006168 level was also increased in ESCC cells (Eca109, KYSE450, KYSE150, and KYSE30), especially in Eca109 and KYSE150 cells (Fig. 1B). Thus, we chose Eca109 and KYSE150 cells for further experiments. Next, we tested the expression of circ_0006168 in parental cells and Taxol-resistant cells. As presented in Fig. 1C, circ_0006168 level in Eca109/Taxol and KYSE150/Taxol cells was higher than that in HET-1A cells and parental cells. Moreover, RNase R treatment assay showed that the expression level of CNOT6L mRNA was significantly reduced, while that of the circ_0006168 showed no obvious change (Fig. 1D), implying the cyclic structure of circ_0006168. These results suggested that circ_0006168 might play an important role in Taxol resistance.

### Knockdown of circ_0006168 increased Taxol sensitivity in Taxol-resistant ESCC cells

**Fig. 2:**
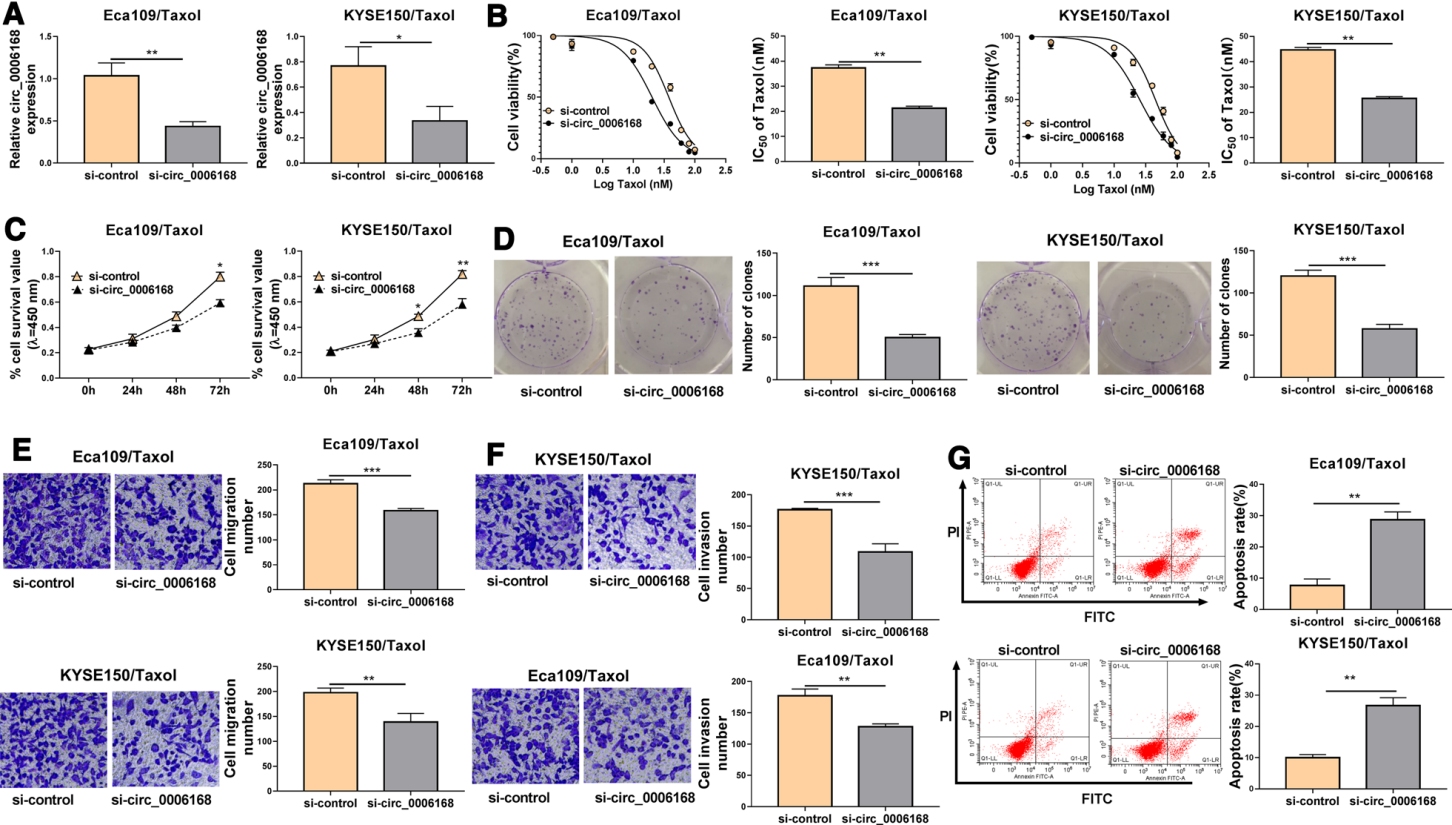
Circ_0006168 silence enhanced Taxol sensitivity of ESCC i*n vivo*. Eca109/Taxol and KYSE150/Taxol were transfected with si-control and si-circ_0006168. **A** The expression of circ_0006168 was measured by qRT-PCR. **B** The cell viability and IC_50_ value of Taxol were measured by CCK-8 assay. **C** CCK-8 assay was employed to evaluate cell viability. **D** Colony formation assay was utilized to evaluate cell colony formation ability. (**E**, **F**) Transwell assay was used to determine cell migration and invasion abilities (× 100). **G** Cell apoptosis was examined using flow cytometry analysis. *P < 0.05, **P < 0.001, ***P < 0.0001

To investigate the effects of circ_0006168 on Taxol sensitivity, Taxol-resistant cells (Eca109/Taxol and KYSE150/Taxol) were transfected with si-control or si-circ_0006168. Knockdown efficiency of circ_0006168 was detected by qRT-PCR. Transfection of si-circ_0006168 could specifically knock down the expression of circ_0006168 in Eca109/Taxol and KYSE150/Taxol (Fig. 2A). CCK-8 assay showed that cell viability and IC_50_ value of Taxol were reduced by downregulating circ_0006168 in Eca109/Taxol and KYSE150/Taxol cells as well as parental cells (Eca109 and KYSE150) (Fig. 2B, Additional file 1: Fig. 1A–D). Next we explored the effect of circ_0006168 overexpression on Taxol toxicity in parental cells. Overexpression efficiency of circ_0006168 was presented in Additional file 2: Fig. 2A, B. We found that circ_0006168 overexpression increased the IC_50_ value of Taxol in Eca109 and KYSE150 cells (Additional file 2: Fig. 2C–F). Meanwhile, CCK-8 assay and colony formation assay indicated that cell viability and colony-forming ability were was impaired by silencing circ_0006168 in Eca109/Taxol and KYSE150/Taxol cells (Fig. 2C, D), suggesting that circ_0006168 silence suppressed proliferation of Eca109/Taxol and KYSE150/Taxol cells. Transwell assay demonstrated that interference of circ_0006168 could reduce migration and invasion of Eca109/Taxol and KYSE150/Taxol cells (Fig. 2E, F). In addition, flow cytometry analysis indicated that circ_0006168 downregulation prominently increased apoptosis of Eca109/Taxol and KYSE150/Taxol cells (Fig. 2G). These data illustrated that circ_0006168 sensitized Eca109/Taxol and KYSE150/Taxol cells to Taxol.

### Circ_0006168 acted as a sponge of miR-194-5p to regulate JMJD1C

**Fig. 3:**
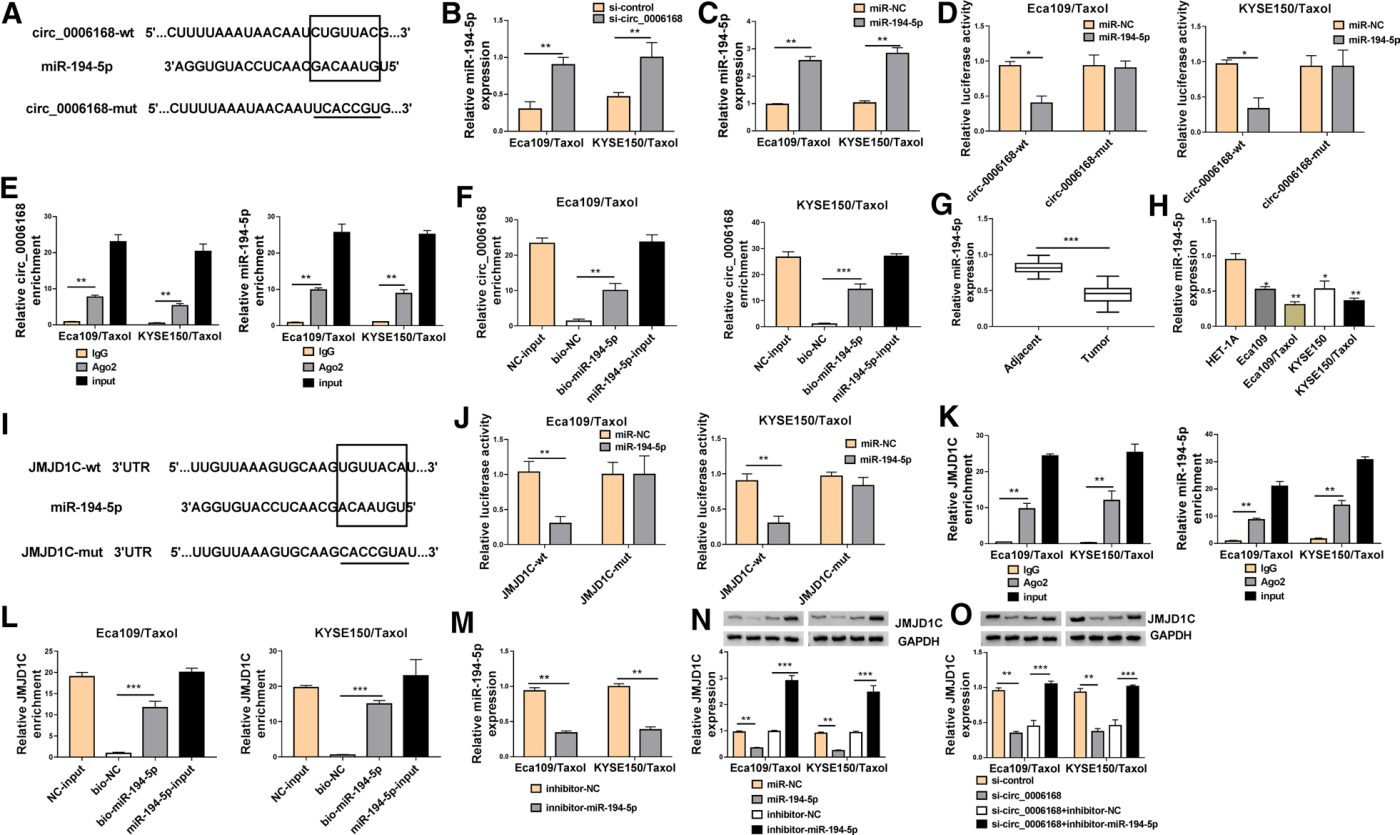
Circ_0006168 regulated JMJD1C expression by acting as a sponge of miR-194-5p. **A** Predicted binding sites between miR-194-5p and circ_0006168 were shown. **B** The expression of miR-194-5p was analyzed by qRT-PCR in Eca109/Taxol and KYSE150/Taxol transfected with si-control and si-circ_0006168. **C** The level of miR-194-5p was examined by qRT-PCR in Eca109/Taxol and KYSE150/Taxol cells transfected with miR-NC or miR-194-5p. **D** Dual-luciferase reporter assay was performed to determine the luciferase activity in Eca109/Taxol and KYSE150/Taxol cells co-transfected with miR-NC or miR-194-5p and circ_0006168-wt or circ_0006168-mut. **E** Circ_0006168 and miR-194-5p enrichment in Eca109/Taxol and KYSE150/Taxol were analyzed by RIP assay. **F** Circ_0006168 enrichment in Eca109/Taxol and KYSE150/Taxol transfected with NC-input, bio-NC, bio-miR-194-5p, or miR-194-5p-input was detected using RNA pull-down assay. **G** The level of miR-194-5p was detected by qRT-PCR in ESCC tissues and adjacent normal tissues. **H** The expression of miR-194-5p was examined by qRT-PCR in HET-1A cells, parental cells (Eca109 and KYSE150), and Taxol-resistant cells (Eca109/Taxol and KYSE150/Taxol). **I** The putative binding sites between miR-194-5p and JMJD1C were predicted by TargetScan. **J** The luciferase activity was determined in Eca109/Taxol and KYSE150/Taxol co-transfected with miR-NC or miR-194-5p and JMJD1C-wt or JMJD1C-mut. **K** JMJD1C and miR-194-5p enrichment in Eca109/Taxol and KYSE150/Taxol were measured by RIP assay. **L** Circ_0006168 enrichment was determined by RNA pull-down assay in Eca109/Taxol and KYSE150/Taxol cells transfected with NC-input, bio-NC, bio-miR-194-5p, or miR-194-5p-input. **M** The expression of miR-194-5p was examined by qRT-PCR Eca109/Taxol and KYSE150/Taxol cells transfected with inhibitor-NC or inhibitor-miR-194-5p. **N** The protein expression of JMJD1C was tested by western blot analysis in Eca109/Taxol and KYSE150/Taxol transfected with miR-NC, miR-194-5p, inhibitor-NC, or inhibitor-miR-194-5p. **O** Western blot assay was carried out to analyze the protein expression of JMJD1C in Eca109/Taxol and KYSE150/Taxol transfected with si-control, si-circ_0006168, si-circ_0006168 + inhibitor-NC, si-circ_0006168 + inhibitor-miR-194-5p. *P < 0.05, **P < 0.001, ***P < 0.0001

CircRNAs are shown to sponge miRNAs and regulate gene expression [25]. To investigate the underlying molecular mechanisms of circ_0006168 in the progression of ESCC, we screened the potential target miRNAs of circ_0006168 by Circinteractome (https://circinteractome.nia.nih.gov/). Results showed that there were potential binding sites between circ_0006168 and miR-194-5p (Fig. 3A), indicating that circ_0006168 might interact with miR-194-5p. Moreover, knockdown of circ_0006168 enhance the expression of miR-194-5p (Fig. 3B). Furthermore, transfection of miR-194-5p increased the expression of miR-194-5p (Fig. 3C), suggesting that miR-194-5p was successfully transfected into Eca109/Taxol and KYSE150/Taxol cells. We constructed dual-luciferase reporter assay, RIP assay and RNA pull-down assay to verify interaction between circ_0006168 and miR-194-5p. The results indicated that the luciferase activity of circ_0006168-wt was reduced by overexpression of miR-194-5p, but the luciferase activity of circ_0006168-mut was not changed (Fig. 3D). RIP assay revealed that both circ_0006168 and miR-194-5p were immunoprecipitated in Ago2 group instead of IgG group (Fig. 3E). Moreover, a considerable amount of circ_0006168 was detected in bio-miR-194-5p group by RNA pull-down assay compared with the bio-NC group (Fig. 3F), suggesting that miR-194-5p could pull down circ_0006168. Next, we detected the expression of miR-194-5p in ESCC tissues ESCC cells, and Taxol-resistant cells. We observed that the expression level of miR-194-5p was significantly lower in ESCC tissues than that in adjacent normal tissues (Fig. 3G). Moreover, miR-194-5p expression was reduced in Eca109/Taxol and KYSE150/Taxol relative to HET-1A cells, and miR-194-5p expression was further decreased in Eca109/Taxol and KYSE150/Taxol (Fig. 3H). Subsequently, we used TargetScan database (http://www.targetscan.org/) to search for the downstream targets of miR-194-5p. As presented in Fig. 3I, there were miR-194-5p binding sites in the 3’UTR of JMJD1C. The results of dual-luciferase reporter assay showed that overexpression of miR-194-5p distinctly degraded the luciferase activity of JMJD1C-wt, but had no significant effect on the luciferase activity of JMJD1C-mut in Eca109/Taxol and KYSE150/Taxol cells (Fig. 3J). Furthermore, RIP assay showed that JMJD1C and miR-194-5p were remarkably enriched in the Ago2 immunoprecipitation compared with the IgG immunoprecipitation (Fig. 3K). Additionally, bio-miR-194-5p led to higher JMJD1C level than treatment of bio-NC group in Eca109/Taxol and KYSE150/Taxol cells (Fig. 3L). Transfection of inhibitor-miR-194-5p reduced the expression of miR-194-5p in Eca109/Taxol and KYSE150/Taxol cells (Fig. 3M). We found that the protein expression of JMJD1C was inhibited by overexpression of miR-194-5p and increased by inhibiting miR-194-5p (Fig. 3N). Next, we explored whether circ_0006168 could regulate the expression of JMJD1C by sponging miR-194-5p. Western blot assay revealed that knockdown of circ_0006168 downregulated the protein expression of JMJD1C, which was restored by downregulating miR-194-5p (Fig. 3O), suggesting that circ_0006168 positively regulated JMJD1C expression by acting as sponge of miR-194-5p.

### Knockdown of JMJD1C enhanced Taxol sensitivity in Taxol-resistant cells

**Fig. 4:**
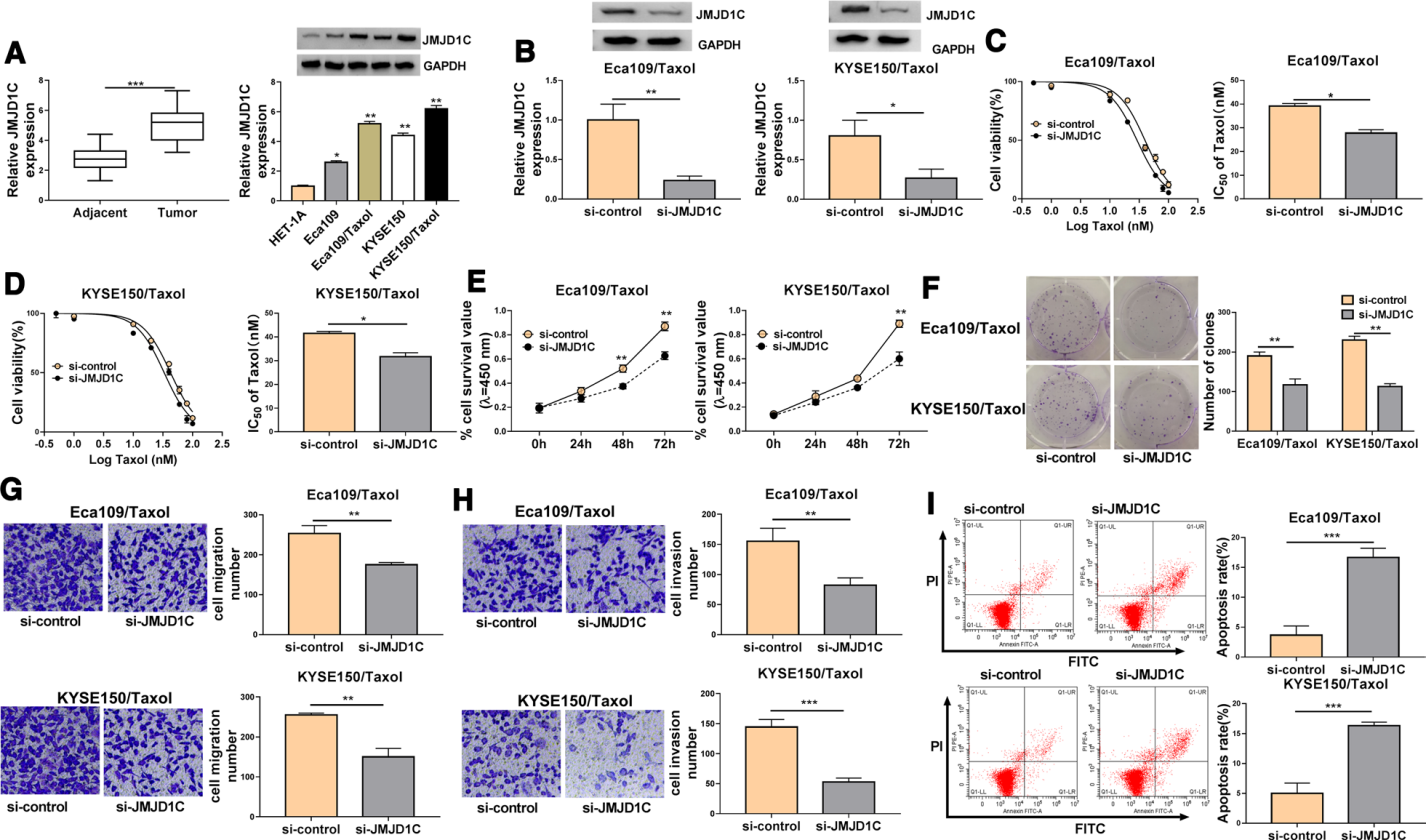
JMJD1C silence improved Taxol sensitivity in Taxol-resistant cells**.** **A** The mRNA expression of JMJD1C was analyzed by qRT-PCR in ESCC tissues and adjacent normal tissues, and western blot assay was performed to measure the protein expression of JMJD1C in HET-1A cells, parental cells (Eca109 and KYSE150), and Taxol-resistant cells (Eca109/Taxol and KYSE150/Taxol). **B**–**I** Eca109/Taxol and KYSE150/Taxol cells were transfected with si-control or si-JMJD1C. **B** The protein expression of JMJD1C was detected by western blot assay. **C**, **D** CCK-8 assay was employed to detect cell viability and IC_50_ value of Taxol. **E** Cell viability was measured using CCK-8 assay. **F** Colony formation assay was utilized to assess cell colony formation ability. **G**, **H** The migration and invasion capacities were evaluated using transwell assay (× 100). **I** Flow cytometry analysis was utilized to determine cell apoptosis. *P < 0.05, **P < 0.001, ***P < 0.0001

To investigate the potential roles of JMJD1C in Taxol resistance, the expression of JMJD1C was detected by qRT-PCR or western blot analysis. The results showed that JMJD1C mRNA expression was increased in ESCC tissues compared to normal tissues (Fig. 4A). Moreover, JMJD1C protein expression was enhanced in Eca109 and KYSE150 cells compared with HET-1A cells, and its expression was markedly higher in Eca109/Taxol and KYSE150/Taxol than that in HET-1A cells (Fig. 4A). JMJD1C protein expression was inhibited by transfection with si-JMJD1C, indicating a high transfection efficiency (Fig. 4B). CCK-8 results showed that JMJD1C knockdown decreased the IC_50_ value of Taxol in Eca109/Taxol and KYSE150/Taxol cells (Fig. 4C, D). Moreover, downregulation of JMJD1C repressed Eca109/Taxol and KYSE150/Taxol cell viability and colony formation ability (Fig. 4E, F). In addition, JMJD1C knockdown suppressed cell migration and invasion and increased apoptosis of Eca109/Taxol and KYSE150/Taxol cells (Fig. 4G–I). All the data suggested that JMJD1C had similar role with circ_0006168 in Taxol-resistant cells.

### Silencing circ_0006168 promoted Taxol sensitivity in Taxol-resistant cells by downregulating JMJD1C

**Fig. 5:**
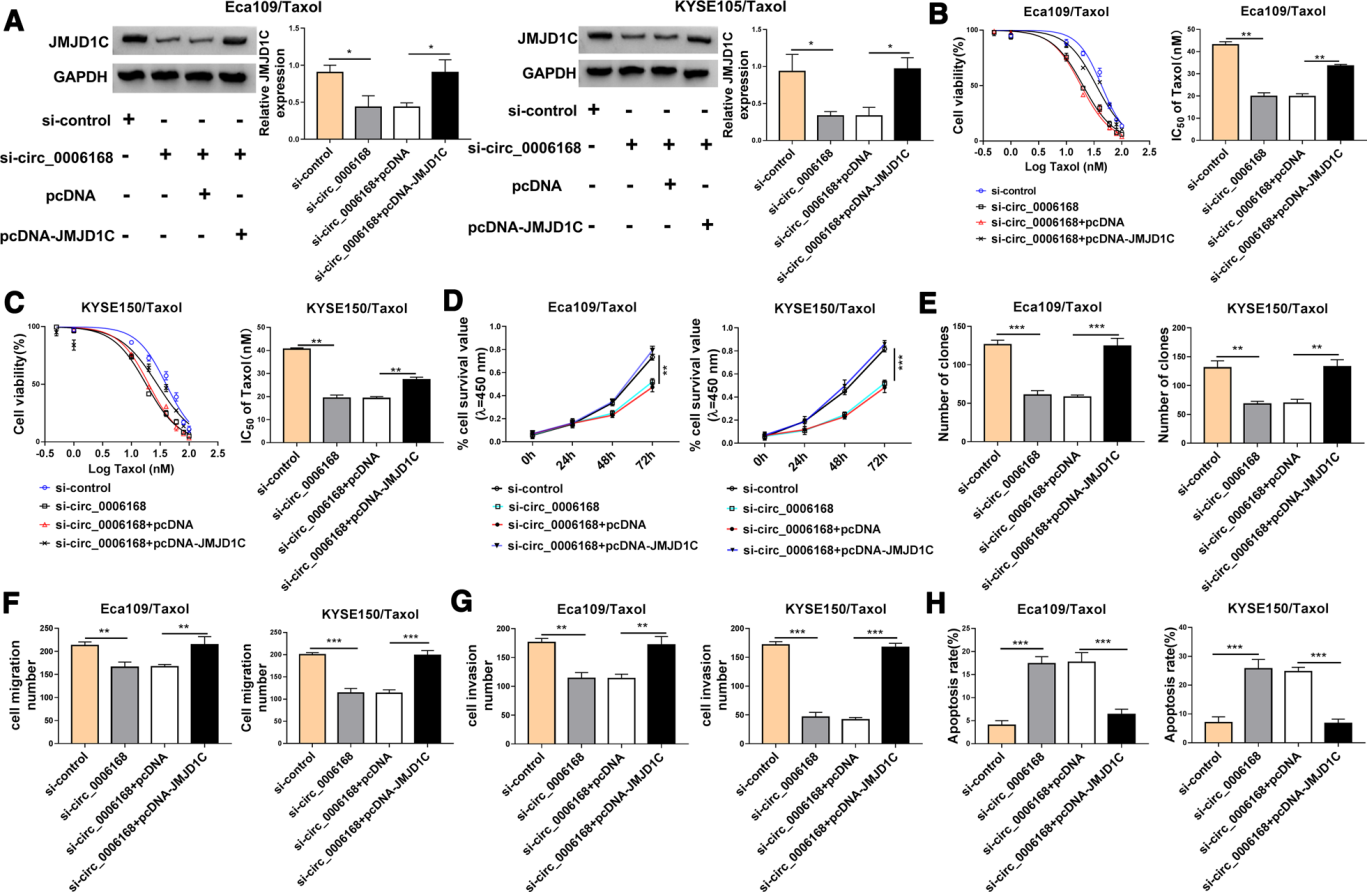
Circ_0006168 regulated Taxol resistance by affecting JMJD1C expression. Eca109/Taxol and KYSE150/Taxol cells were transfected with si-control, si-circ_0006168, si-circ_0006168 + pcDNA, or si-circ_0006168 + pcDNA-JMJD1C. **A** Western blot assay was used to analyze the protein abundance of JMJD1C. **B**, **C** Cell viability and IC_50_ value of Taxol were determined by CCK-8 analysis. **D**, **E** CCK-8 assay and colony formation assay were employed to evaluate cell proliferation. **F**, **G** Cell migration and invasion abilities were assessed by transwell assay. **H** Flow cytometry analysis was applied to detect cell apoptosis. *P < 0.05, **P < 0.001, ***P < 0.0001

Rescue experiments were conducted to evaluate the regulatory effects of circ_0006168/JMJD1C axis on Taxol sensitivity. Western blot assay indicted that circ_0006168 knockdown reduced the protein expression of JMJD1C, which was rescued by upregulating JMJD1C (Fig. 5A). Moreover, the effect of circ_0006168 downregulation on reduction of IC_50_ value of Taxol was abolished by elevating JMJD1C in Eca109/Taxol and KYSE150/Taxol cells (Fig. 5B, C). In addition, overexpression of JMJD1C reversed the inhibitory effects of circ_0006168 silence on cell viability, colony formation, migration, and invasion in Eca109/Taxol and KYSE150/Taxol cells (Fig. 5D–G). Furthermore, JMJD1C upregulation attenuated si-circ_0006168-induced apoptosis in Eca109/Taxol and KYSE150/Taxol cells (Fig. 5H). These results indicated that circ_0006168 exerted its functions by regulating JMJD1C in ESCC cells.

### Circ_0006168 downregulation inhibited tumor growth in vivo

**Fig. 6:**
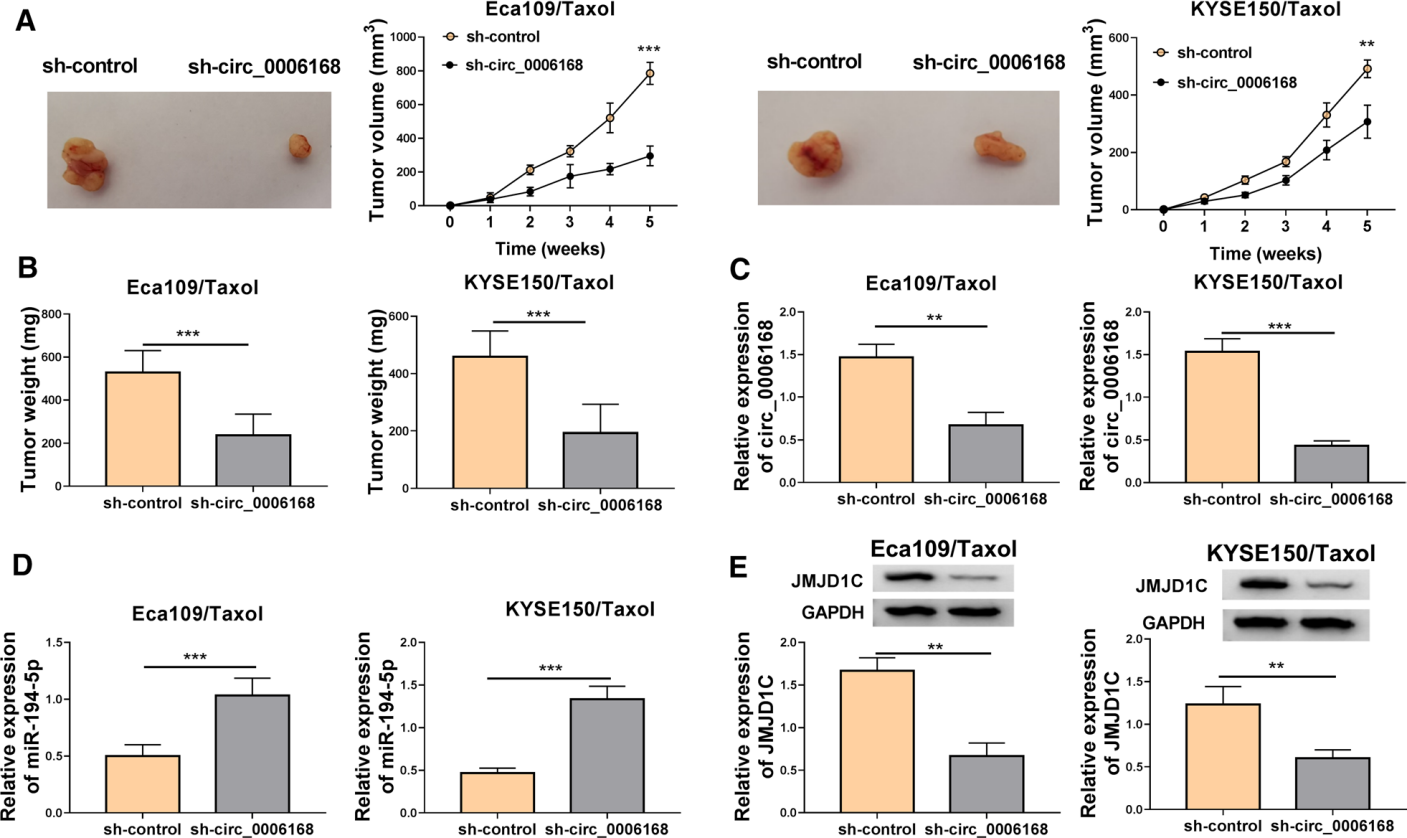
Circ_0006168 knockdown repressed tumor growth in vivo. Eca109/Taxol and KYSE150/Taxol cells transfected with sh-control or sh-circ_0006168 were injected into nude mice to establish mice xenograft model. **A**, **B** Tumor volume and weight were measured. **C**, **D** The expression levels of circ_0006168 and miR-194-5p were measured by qRT-PCR in the collected tissues. **E** Western blot assay was used to analyze the protein expression of JMJD1C in the collected tissues. **P < 0.001, ***P < 0.0001

To elucidate the effect of circ_0006168 in vivo, Eca109/Taxol and KYSE150/Taxol cells transfected with sh-NC or sh-circ_0006168 were subcutaneously injected into nude mice. Knockdown of circ_0006168 inhibited tumor volume and weight in Eca109/Taxol and KYSE150/Taxol xenograft models (Fig. 6A, B). Moreover, the expression levels of circ_0006168, miR-194-5p and JMJD1C were measured in tumor tissues. As displayed in Fig. 6C, D, circ_0006168 deficiency reduced circ_0006168 expression and enhanced miR-194-5p expression in tissues from Eca109/Taxol and KYSE150/Taxol xenograft mice. In addition, JMJD1C protein expression was decreased in sh-circ_0006168 group compared with that in sh-control group (Fig. 6E). These data indicated that circ_0006168 silence repressed tumor growth by upregulating miR-194-5p and downregulating JMJD1C.

## Discussion

ESCC is a common malignant tumor of the digestive system [26]. Chemotherapy, including Taxol, is a promising treatment for patients with advanced ESCC who cannot undergo resection [22]. The development of chemoresistance has become a major obstacle limiting the efficacy of chemotherapy [27]. Growing evidence has certified that circRNAs function as crucial regulators in different cellular physiological processes [28, 29]. Compared to miRNAs and lncRNAs, circRNAs are highly abundant, stable, and specifically expressed [30, 31]. Thus, circRNAs have become a hot topic in carcinoma research. Recently, although more and more reports have suggested that some circRNAs are involved in the progression of multiple cancers and chemoresistance [32, 33], the functions of the majority of circRNAs remain unclear. Herein, we investigated the roles and regulatory mechanism of circ_0006168 in Taxol resistance of ESCC.

Previous studies have shown that some circRNAs are associated with the progression and development of ESCC [34–36]. More importantly, Xie et al*.* declared that circ_0006168 was overexpressed in EC tumors and cells, and inhibition of circ_0006168 attenuated cell growth migration, invasion, and glycolysis in EC via modulating miR-384/RBBP7 axis [37]. Moreover, Shi e*t al.* also declared that high level of circ_0006168 level was positively correlated with TNM stage and lymph node metastasis in the patients with ESCC, and circ_0006168 facilitated the growth and metastasis of ESCC cells via sponging miR-100 and regulating mTOR [16]. Nevertheless, the influence of circ_0006168 on Taxol resistance of ESCC has not been clarified. In this research, we observed an obvious increase of circ_0006168 expression not only in ESCC tissues and cells but also in Taxol-resistant cells. In vitro functional experiments revealed that circ_0006168 downregulation repressed cell proliferation, invasion and migration as well as accelerated apoptosis in Taxol-resistant cells. Taken together, these findings implicated that circ_0006168 promoted Taxol resistance in ESCC.

Mechanistically, circRNAs can exert their functions in human malignant tumors via competitively binding to miRNAs and regulating the expression of target genes [38]. For instance, hsa_circ_0030018 served as a miR-599 sponge to facilitate EC progression through upregulating ENAH expression [39]. CircRNA circ-Foxo3 inhibited ESCC progression via sponging miR-23a and upregulating PTEN [40]. In this research, we confirmed that miR-194-5p directly bound to circ_0006168, which was a key miRNA that targeted multiple genes. MiR-194-5p was reported to be involved in regulating chemoresistance, including Taxol resistance [22, 41]. Previous studies have proven that miR-194-5p payed a tumor-suppressive role and downregulated in many cancers, including ESCC [21, 42, 43]. Consistent with these reports, we uncovered that miR-194-5p was obviously reduced in ESCC tissues, ESCC cells, and Taxol-resistant cells. Next, TargetScan database was utilized to search for the downstream targets of miR-194-5p. We revealed that JMJD1C acted as a downstream target of miR-194-5p, and circ_0006168 could positively modulate JMJD1C expression via sponging miR-194-5p. JMJD1C was reported to function as a tumor facilitator in some cancers by promoting cancer proliferation and inhibiting apoptosis [44, 45]. Ek et al*.* demonstrated that JMJD1C might be associated with the risk of EC [46]. Cai et al*.* proved that JMJD1C level was positively correlated with the TNM stage, and knockdown of JMJD1C inhibited EC cell proliferation by regulating YAP1 signaling [24]. However, the biological functions of JMJD1C in Taxol resistance of ESCC have not been illustrated. In our study, we confirmed that JMJD1C showed higher level in ESCC tissues, ESCC cells, and Taxol-resistant cells. Moreover, JMJD1C interference inhibited cell growth, invasion and migration and facilitated apoptosis in Taxol-resistant cells, indicating that JMJD1C knockdown could attenuate Taxol resistance. Rescue assays proved that JMJD1C overexpression countervailed the promotive role of Taxol sensitivity caused by circ_0006168 knockdown, suggesting that circ_0006168 contributed to Taxol resistance of ESCC cells by upregulating JMJD1C. In addition, in vivo experiments revealed that circ_0006168 deficiency suppressed tumor growth by increasing miR-194-5p and decreasing JMJD1C, implying that circ_0006168 silence might improve Taxol sensitivity in vivo. Although circ_0006168 knockdown inhibits tumor growth in vivo, this effect does not necessarily translate into meaningful clinical benefits. Therefore, it is necessary to further study the role of circ_0006168 in clinical practice. Moreover, the circRNAs/miRNAs/mRNAs regulatory networks are very complicated, thus, the detailed physiological mechanisms for the effects of circ_0006168 in ESCC and Taxol resistance need further exploration.

**Fig. 7:**
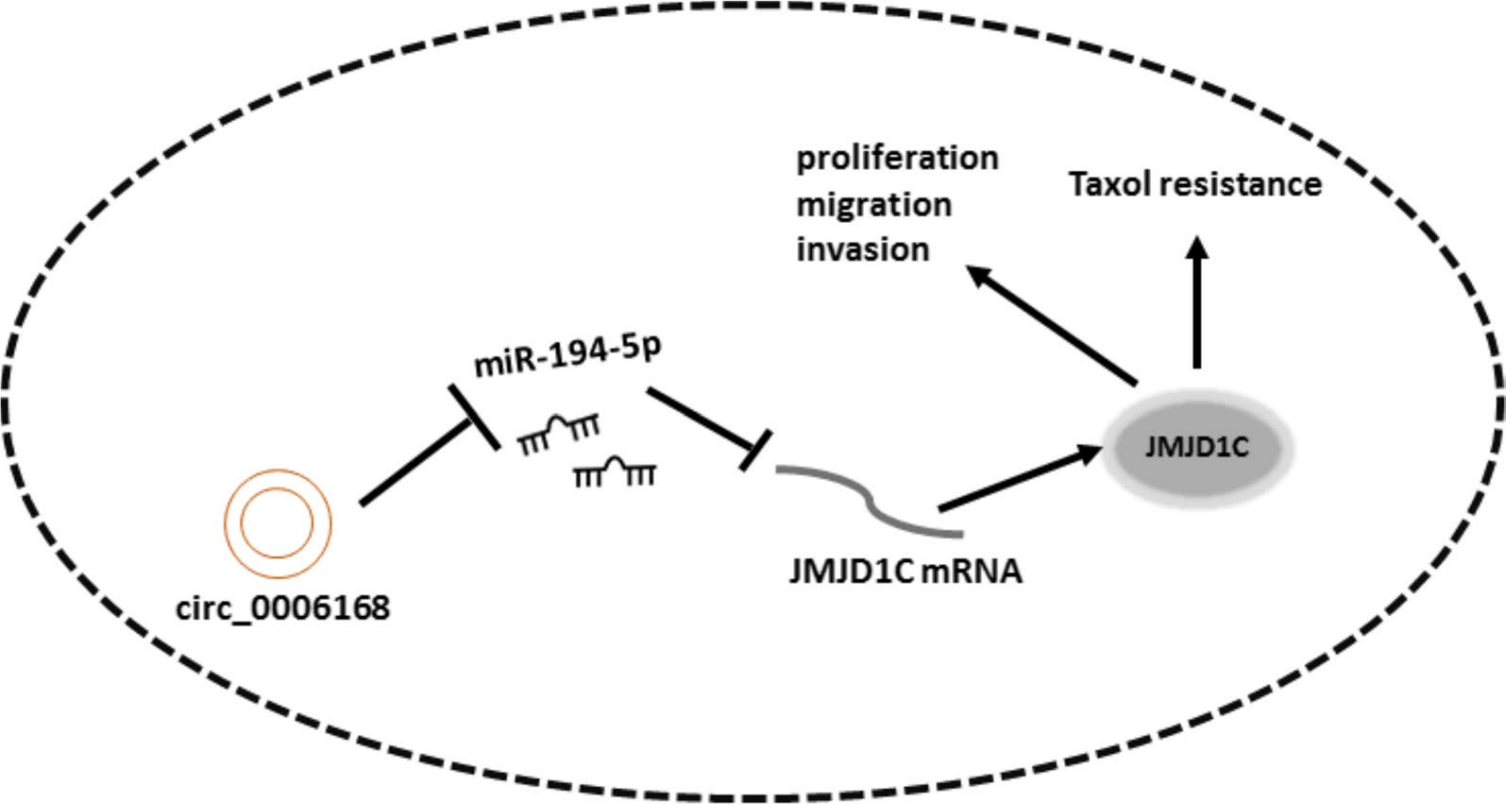
Schematic diagram of the mechanism by which the circ_0006168/miR-194-5p/JMJD1C regulates cell proliferation, migration, invasion, and Taxol resistance in Taxol-resistant ESCC cells

In conclusion, interference of circ_0006168 restrained cell proliferation, invasion and migration and accelerated apoptosis of Taxol-resistant cells in vitro, and inhibited tumor growth in vivo via sponging miR-194-5p and regulating JMJD1C (Fig. 7). It provided experimental basis for studying tumor resistance and further targeted therapy.

## Supplementary Information

**Additional file 1: Figure S1.** Circ_0006168 knockdown enhanced Taxol toxicity in parental cells. (**A–D**) Cell viability and IC_50_ value of Taxol were determined by CCK-8 analysis in Eca109 and KYSE150 cells transfected with si-NC or si-circ_0006168 and then treated with different doses of Taxol. **P < 0.001.

**Additional file 2: Figure S2.** Circ_0006168 overexpression inhibited Taxol toxicity in parental cells. **A**, **B** The expression of circ_0006168 was detected by qRT-PCR in Eca109 and KYSE150 cells transfected with pcD-ciR or circ_0006168. **C**–**F** Cell viability and IC_50_ value of Taxol were measured by CCK-8 assay in Eca109 and KYSE150 cells transfected with pcD-ciR or circ_0006168 and then treated with different doses of Taxol. *P* < 0.05, **P < 0.001.

## Data Availability

The datasets used and/or analysed during the current study are available from the corresponding author on reasonable request.

## Abbreviations

ESCC: Esophageal squamous cell carcinoma
JMJD1C: Jumonji domain containing 1C
CCK-8: Cell counting Kit-8
EC: Esophageal cancer
CNOT6L: Complex subunit 6 like

## References

[1] Bray F, Ferlay J, Soerjomataram I, Siegel RL, Torre LA, Jemal A, Global cancer statistics (2018). GLOBOCAN estimates of incidence and mortality worldwide for 36 cancers in 185 countries. CA Cancer J Clin.

[2] Kato K, Cho BC, Takahashi M, Okada M, Lin CY, Chin K, Kadowaki S, Ahn MJ, Hamamoto Y, Doki Y, Yen CC, Kubota Y, Kim SB, Hsu CH, Holtved E, Xynos I, Kodani M, Kitagawa Y (2019). Nivolumab versus chemotherapy in patients with advanced oesophageal squamous cell carcinoma refractory or intolerant to previous chemotherapy (ATTRACTION-3): a multicentre, randomised, open-label, phase 3 trial. Lancet Oncol.

[3] Arnold M, Soerjomataram I, Ferlay J, Forman D (2015). Global incidence of oesophageal cancer by histological subtype in 2012. Gut.

[4] Huang F, Yu S (2016). Esophageal cancer: Risk factors, genetic association, and treatment. Asian J Surg.

[5] Abnet CC, Arnold M, Wei W (2017). Epidemiology of esophageal squamous cell carcinoma. Gastroenterology.

[6] Feng W, Xiaoyan X, Xuan Y, Xiangke L, Zichang Y, Ran Z, Liuxing W, Qingxia F (2015). Silencing stathmin-modulating efficiency of chemotherapy for esophageal squamous cell cancer with paclitaxel. Cancer Gene Ther.

[7] Miyawaki Y, Nakajima Y, Kawada K, Okada T, Tokairin Y, Kawano T (2017). Efficacy of docetaxel, cisplatin, and 5-fluorouracil chemotherapy for superficial esophageal squamous cell carcinoma. Dis Esophagus.

[8] Jeck WR, Sharpless NE (2014). Detecting and characterizing circular RNAs. Nat Biotechnol.

[9] Guo Y, Yang J, Huang Q, Hsueh C, Zheng J, Wu C, Chen H, Zhou L (2019). Circular RNAs and their roles in head and neck cancers. Mol Cancer.

[10] Memczak S, Jens M, Elefsinioti A, Torti F, Krueger J, Rybak A, Maier L, Mackowiak SD, Gregersen LH, Munschauer M (2013). Circular RNAs are a large class of animal RNAs with regulatory potency. Nature.

[11] Shang Q, Yang Z, Jia R, Ge S (2019). The novel roles of circRNAs in human cancer. Mol Cancer.

[12] Wang Q, Zhang Q, Sun H, Tang W, Yang L, Xu Z, Liu Z, Jin H, Cao X (2019). Circ-TTC17 Promotes proliferation and migration of esophageal squamous cell carcinoma. Dig Dis Sci.

[13] Song H, Xu D, Shi P, He B, Li Z, Ji Y, Agbeko CK, Wang J (2019). Upregulated circ RNA hsa_circ_0000337 promotes cell proliferation, migration, and invasion of esophageal squamous cell carcinoma. Cancer Manag Res.

[14] Xu N, Chen S, Liu Y, Li W, Liu Z, Bian X, Ling C, Jiang M (2018). Profiles and bioinformatics analysis of differentially expressed circrnas in Taxol-resistant non-small cell lung cancer cells. Cell Physiol Biochem.

[15] Liu YY, Zhang LY, Du WZ (2019). Biosci Rep.

[16] Shi Y, Guo Z, Fang N, Jiang W, Fan Y, He Y, Ma Z, Chen Y (2019). hsa_circ_0006168 sponges miR-100 and regulates mTOR to promote the proliferation, migration and invasion of esophageal squamous cell carcinoma. Biomed Pharmacother.

[17] Li X, Feng Y, Yang B, Xiao T, Ren H, Yu X, Li L, Li M, Zhang W (2020). A novel circular RNA, hsa_circ_0030998 suppresses lung cancer tumorigenesis and Taxol resistance by sponging miR-558. Mol Oncol.

[18] Li X, Yang B, Ren H, Xiao T, Zhang L, Li L, Li M, Wang X, Zhou H, Zhang W (2019). Hsa_circ_0002483 inhibited the progression and enhanced the Taxol sensitivity of non-small cell lung cancer by targeting miR-182-5p. Cell Death Dis.

[19] Bach DH, Lee SK, Sood AK (2019). Circular RNAs in Cancer. Mol Ther Nucleic acids.

[20] Qu F, Cao P (2018). Long noncoding RNA SOX2OT contributes to gastric cancer progression by sponging miR-194-5p from AKT2. Exp Cell Res.

[21] Cui G, Liu D, Li W, Li Y, Liang Y, Shi W, Zhao S (2017). Original Research: miR-194 inhibits proliferation and invasion and promotes apoptosis by targeting KDM5B in esophageal squamous cell carcinoma cells. Exp Biol Med (Maywood).

[22] Nakamura K, Sawada K, Miyamoto M, Kinose Y, Yoshimura A, Ishida K, Kobayashi M, Shimizu A, Nakatsuka E, Hashimoto K, Mabuchi S, Kimura T (2019). Downregulation of miR-194-5p induces paclitaxel resistance in ovarian cancer cells by altering MDM2 expression. Oncotarget.

[23] Watanabe S, Watanabe K, Akimov V, Bartkova J, Blagoev B, Lukas J, Bartek J (2013). JMJD1C demethylates MDC1 to regulate the RNF8 and BRCA1-mediated chromatin response to DNA breaks. Nat Struct Mol Biol.

[24] Cai Y, Fu X, Deng Y (2017). Histone demethylase JMJD1C regulates esophageal cancer proliferation Via YAP1 signaling. Am J Cancer Res.

[25] Kulcheski FR, Christoff AP, Margis R (2016). Circular RNAs are miRNA sponges and can be used as a new class of biomarker. J Biotechnol.

[26] Alsop BR, Sharma P (2016). Esophageal Cancer. Gastroenterol Clin North Am.

[27] Vasan N, Baselga J, Hyman DM (2019). A view on drug resistance in cancer. Nature.

[28] Arnaiz E, Sole C, Manterola L, Iparraguirre L, Otaegui D, Lawrie CH (2019). CircRNAs and cancer: Biomarkers and master regulators. Semin Cancer Biol.

[29] Zhang HD, Jiang LH, Sun DW, Hou JC, Ji ZL (2018). CircRNA: a novel type of biomarker for cancer. Breast Cancer.

[30] Vidal AF, Sandoval GT, Magalhães L, Santos SE, Ribeiro-dos-Santos Â (2016). Circular RNAs as a new field in gene regulation and their implications in translational research. Epigenomics.

[31] Li X, Yang L, Chen LL (2018). The biogenesis, functions, and challenges of circular RNAs. Mol Cell.

[32] Guo C, Wang H, Jiang H, Qiao L, Wang X (2020). Circ_0011292 enhances paclitaxel resistance in non-small cell lung cancer by regulating miR-379–5p/TRIM65 Axis. Cancer Biother Radiopharm.

[33] Wu Q, Wang H, Liu L, Zhu K, Yu W, Guo J (2020). Hsa_circ_0001546 acts as a miRNA-421 sponge to inhibit the chemoresistance of gastric cancer cells via ATM/Chk2/p53-dependent pathway. Biochem Biophys Res Commun.

[34] Cao S, Chen G, Yan L, Li L, Huang X (2018). Contribution of dysregulated circRNA_100876 to proliferation and metastasis of esophageal squamous cell carcinoma. Onco Targets Ther.

[35] Xia W, Qiu M, Chen R, Wang S, Leng X, Wang J, Xu Y, Hu J, Dong G, Xu PL, Yin R (2016). Circular RNA has_circ_0067934 is upregulated in esophageal squamous cell carcinoma and promoted proliferation. Sci Rep.

[36] Zheng B, Wu Z, Xue S, Chen H, Zhang S, Zeng T, Xu G, Wu W, Zheng W, Chen C (2019). hsa_circRNA_100873 upregulation is associated with increased lymphatic metastasis of esophageal squamous cell carcinoma. Oncol Lett.

[37] Xie ZF, Li HT, Xie SH, Ma M (2020). Circular RNA hsa_circ_0006168 contributes to cell proliferation, migration and invasion in esophageal cancer by regulating miR-384/RBBP7 axis via activation of S6K/S6 pathway. Eur Rev Med Pharmacol Sci.

[38] Greene J, Baird AM, Brady L, Lim M, Gray SG, McDermott R, Finn SP (2017). Circular RNAs: biogenesis function and role in human diseases. Front Mol Biosci.

[39] Wang C, Tang D, Wang H, Hu G, Hu S, Li L, Min B, Wang Y (2019). Circular RNA hsa_circ_0030018 acts as a sponge of miR-599 to aggravate esophageal carcinoma progression by regulating ENAH expression. J Cell Biochem.

[40] Xing Y, Zha WJ, Li XM, Li H, Gao F, Ye T, Du WQ, Liu YC (2020). Circular RNA circ-Foxo3 inhibits esophageal squamous cell cancer progression via the miR-23a/PTEN axis. J Cell Biochem.

[41] Wu J, Zhang L, Wu S, Yi X, Liu Z (2020). miR-194-5p inhibits SLC40A1 expression to induce cisplatin resistance in ovarian cancer. Pathol Res Pract.

[42] Wang Y, Sun G, Wang C, Guo W, Tang Q, Wang M (2018). MiR-194-5p inhibits cell migration and invasion in bladder cancer by targeting E2F3. J BUON.

[43] Wang Y, Yang L, Chen T, Liu X, Guo Y, Zhu Q, Tong X, Yang W, Xu Q, Huang D, Tu K (2019). A novel lncRNA MCM3AP-AS1 promotes the growth of hepatocellular carcinoma by targeting miR-194-5p/FOXA1 axis. Mol Cancer.

[44] Chen C, Aihemaiti M, Zhang X, Qu H, Sun QL, He QS, Yu WB (2018). Downregulation of histone demethylase JMJD1C inhibits colorectal cancer metastasis through targeting ATF2. Am J Cancer Res.

[45] Sroczynska P, Cruickshank VA, Bukowski JP, Miyagi S, Bagger FO, Walfridsson J, Schuster MB, Porse B, Helin K (2014). shRNA screening identifies JMJD1C as being required for leukemia maintenance. Blood.

[46] Ek WE, Lagergren K, Cook M, Wu AH, Abnet CC, Levine D, Chow WH, Bernstein L, Risch HA, Shaheen NJ, Bird NC, Corley DA, Hardie LJ, Fitzgerald RC, Gammon MD, Romero Y, Liu G, Ye W, Vaughan TL, MacGregor S, Whiteman DC, Westberg L, Lagergren J (2016). Polymorphisms in genes in the androgen pathway and risk of Barrett's esophagus and esophageal adenocarcinoma. Int J Cancer.

