# Supplementary figures and images for: Circular RNA circ_0006168 enhances Taxol resistance in esophageal squamous cell carcinoma by regulating miR-194-5p/JMJD1C axis
Source: Cancer Cell Int. 2021 May 22;21:273. doi: 10.1186/s12935-021-01984-y (PMC8141117; doi:10.1186/s12935-021-01984-y)

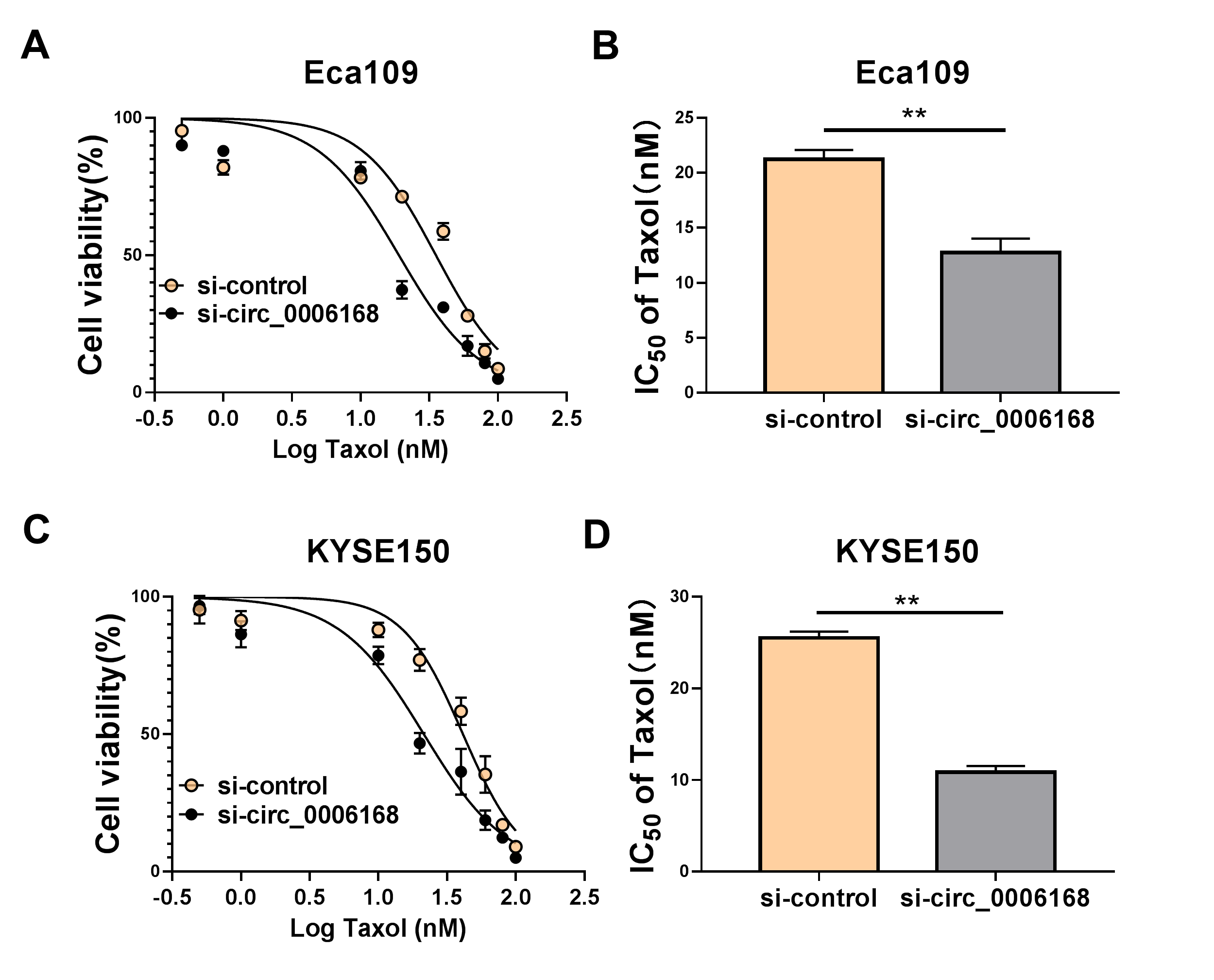

Supplement: Supplementary file 1 — Additional file 1: Figure S1. Circ_0006168 knockdown enhanced Taxol toxicity in parental cells. (A–D) Cell viability and IC50 value of Taxol were determined by CCK-8 analysis in Eca109 and KYSE150 cells transfected with si-NC or si-circ_0006168 and then treated with different doses of Taxol. **P < 0.001. [file 12935_2021_1984_MOESM1_ESM.tif]

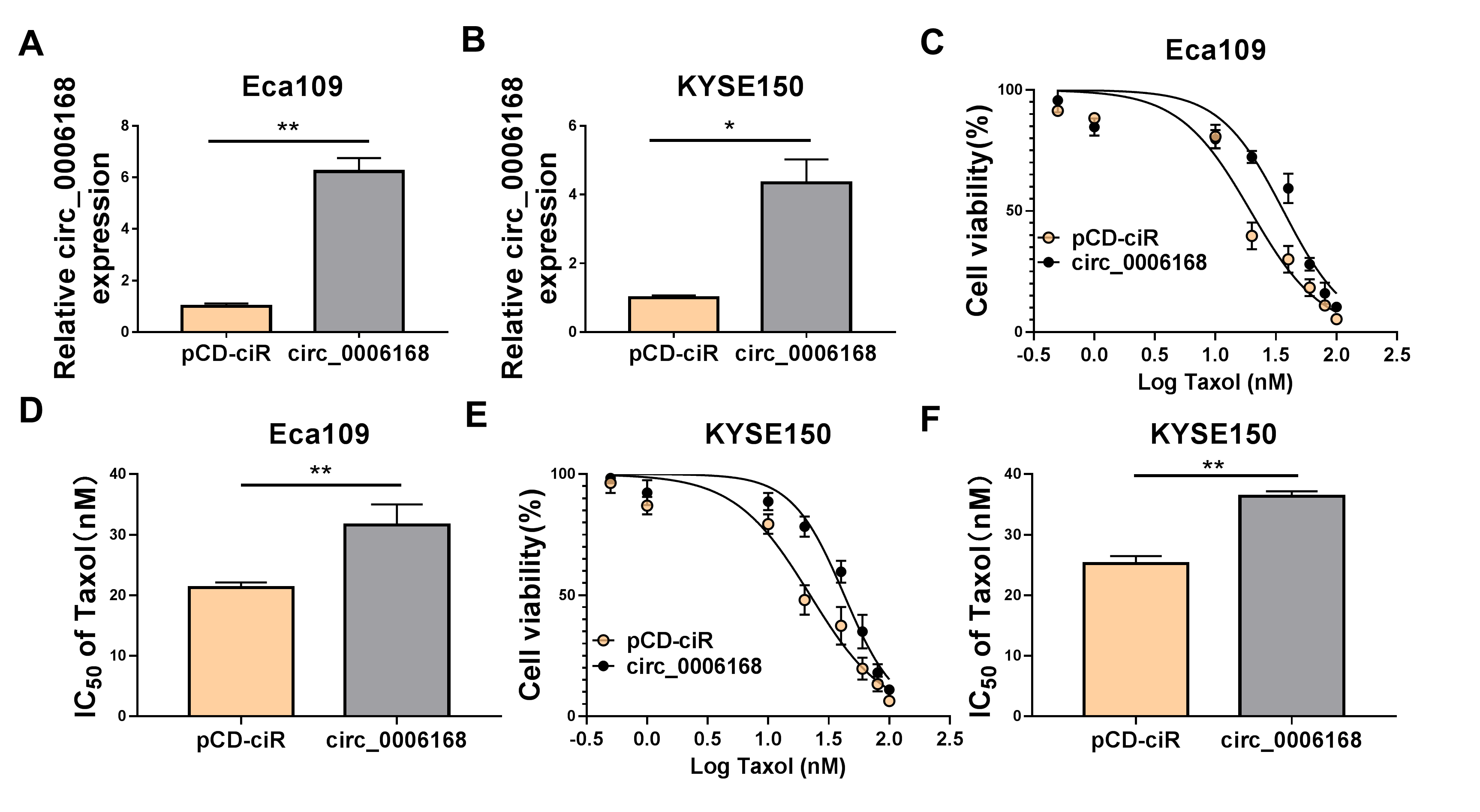

Supplement: Supplementary file 2 — Additional file 2: Figure S2. Circ_0006168 overexpression inhibited Taxol toxicity in parental cells. A, B The expression of circ_0006168 was detected by qRT-PCR in Eca109 and KYSE150 cells transfected with pcD-ciR or circ_0006168. C–F Cell viability and IC50 value of Taxol were measured by CCK-8 assay in Eca109 and KYSE150 cells transfected with pcD-ciR or circ_0006168 and then treated with different doses of Taxol. P < 0.05, **P < 0.001. [file 12935_2021_1984_MOESM2_ESM.tif]
